# How host–guest interaction types shape value co-creation in urban tourism: The moderating role of AI technology stimuli

**DOI:** 10.1371/journal.pone.0348028

**Published:** 2026-04-28

**Authors:** Guanxi Chen, Jiajun Chen

**Affiliations:** 1 NingboTech University, School of Design, Ningbo, China; 2 Department of Business Administration, Joongbu University, Goyang, Korea; 3 Nantong Institute of Technology, School of Business, Nantong‌‌, China; Sichuan Agricultural University, CHINA

## Abstract

This study examines how different types of host–guest interaction relate to tourists’ value co-creation intention in urban tourism and whether artificial intelligence technology stimuli moderate these relationships. Grounded theory was used to identify the main constructs and relationships. Survey data were then analyzed using partial least squares structural equation modeling, and fuzzy-set qualitative comparative analysis was conducted to identify configurations associated with high value co-creation intention. The results showed that tourist–government interaction and tourist–resident interaction were positively related to tourist empowerment, and tourist empowerment was positively related to value co-creation intention. Neither tourist–government interaction nor tourist–resident interaction had a direct relationship with value co-creation intention. Artificial intelligence technology stimuli moderated the paths from tourist–government interaction to tourist empowerment and from tourist–government interaction to value co-creation intention. No moderating effect was found for the tourist–resident interaction paths. The fuzzy-set qualitative comparative analysis identified multiple configurations associated with high value co-creation intention.

## 1 Introduction

The diffusion of smart tourism has accelerated the adoption of artificial intelligence (AI) in destination management and tourist services, reshaping how tourists access information, communicate with local actors, and participate in destination experiences [1]. In this context, value co-creation is increasingly understood as an interaction-based and resource-integrative process in which tourists do not merely consume pre-designed services, but actively participate in shaping service experiences and destination value together with multiple stakeholders [2]. Such a view implies that tourism destinations should be understood not simply as service delivery settings, but as dynamic service ecosystems in which value emerges through interaction, resource integration, and use-in-context [3].

Existing research on tourism value co-creation has developed along several related strands. First, studies of tourist experience emphasize tourists as active agents who co-create value through engagement with local environments, communities, and cultural resources [4], and through the use of personal expertise and situational involvement to shape unique experiences [5]. Second, research in management and marketing highlights co-innovation between tourism providers and tourists as a strategic approach to adapting offerings and strengthening destination branding [6]. Third, work on residents’ attitudes and behaviors suggests that host–guest interaction—across cognitive, emotional, and behavioral dimensions—affects residents’ perceptions of tourism development and their willingness to participate in co-creation practices [7]. Related studies also indicate that platform identification, resource sharing, and prior experience can predict tourists’ co-creation behavior and promote loyalty and sustained participation in platform ecosystems [8]. While these studies have substantially advanced understanding of tourism co-creation, they tend to treat interaction as a broadly favorable antecedent, rather than critically examining whether different forms of host–guest interaction activate co-creation through distinct mechanisms and under different technological conditions.

This limitation is particularly salient in smart tourism contexts, where interaction is no longer confined to face-to-face encounters between tourists and local residents, but increasingly unfolds across digitally mediated, institutionally organized, and platform-supported relational settings [9]. Under such conditions, host–guest interaction should not be viewed as a homogeneous phenomenon. Rather, different interaction forms may provide different kinds of operant and operand resources, shape tourists’ sense of participation in different ways, and ultimately generate unequal implications for value co-creation [10]. However, current research has not sufficiently theorized this heterogeneity. In particular, although host–guest interaction has often been recognized as a relevant context for co-creation [11], prior‌‌ studies seldom clarify which interaction types are more likely to move tourists from passive service recipients to active contributors, especially in urban tourism systems increasingly mediated by smart platforms and public interfaces.

A second unresolved issue concerns the psychological mechanism through which interaction translates into co-creation outcomes. Existing studies have frequently relied on consumer-behavior frameworks such as the stimulus–organism–response (SOR) model [12], yet relatively less attention has been paid to how interaction generates an internal sense of agency that makes co-creation psychologically actionable and meaningful. In co-creation settings, tourists do not become active contributors simply because interaction occurs; rather, they are more likely to engage in co-creation when interaction enhances their perceived ability to express preferences, influence processes, and participate meaningfully in the destination service system [13]. This suggests that customer empowerment is not merely an auxiliary psychological variable, but a crucial explanatory mechanism through which interaction is converted into participatory intention [14].

A third gap relates to the role of AI in host–guest interaction. Although AI tools such as chatbots, personalized recommendation systems, and smart guidance services are increasingly deployed to improve responsiveness, efficiency, and experiential support in tourism [15], their role in value co-creation remains insufficiently specified. Existing studies often imply that AI uniformly enhances tourist participation and service experience [16], yet this assumption may overlook the possibility that the effectiveness of AI is contingent on the type of interaction in which it is embedded. AI may be more easily aligned with institutional and procedural forms of interaction that rely on information processing, service coordination, and feedback responsiveness, whereas its role may be more limited in affective and culturally embedded interactions that depend on emotional resonance, mutual recognition, and everyday social warmth [17]. In this sense, AI technology stimuli should be understood not simply as a general technological backdrop, but as a context-sensitive boundary condition whose amplifying effect may vary across interaction types.

To address these gaps, this study examines how host–guest interaction types shape tourists’ willingness to co-create value in urban tourism through an indirect and mechanism-based pathway rather than through uniformly assumed direct effects. Specifically, the study focuses on two interaction types that are especially salient in China’s smart tourism governance context: tourist–government interaction, which refers to institutional interaction through smart platforms and public service mechanisms, and tourist–resident interaction, which refers to affective and culturally embedded interaction rooted in resident attitudes, local expression, and lifestyle contact [18]. This distinction is theoretically important because it moves beyond treating host–guest interaction as a single relational category and instead recognizes that different host actors may offer different participation conditions, resource access points, and meaning-making processes.

Building on this distinction, the study argues that host–guest interaction is more likely to foster co-creation willingness by enhancing tourists’ perceived customer empowerment, understood as their perceived agency, influence, and participatory capability within the destination service system [12]. In other words, interaction does not automatically generate co-creation intention; rather, it creates conditions under which tourists may come to see themselves as capable and legitimate contributors to destination value formation. Meanwhile, AI technology stimuli may function as a contextual amplifier that strengthens—or fails to strengthen—this interaction–empowerment–co-creation linkage depending on the interaction type. This perspective helps reposition AI not as a universally beneficial tool, but as a differentiated enabler whose effects depend on the social and institutional structure of interaction.

Accordingly, this study addresses three research questions: (1) What types of host–guest interactions emerge in smart tourism contexts, and how are they associated with tourists’ willingness to co-create value? (2) Through what mediating mechanism does host–guest interaction influence co-creation willingness? (3) How do AI technology stimuli moderate the effects of different interaction types within this process? By answering these questions, this study contributes to the literature in three ways. First, it differentiates host–guest interaction into distinct relational forms rather than treating it as a homogeneous construct. Second, it clarifies customer empowerment as a key psychological mechanism through which interaction is translated into co-creation intention. Third, it specifies AI technology stimuli as a contingent boundary condition, thereby offering a more nuanced understanding of how intelligent technologies reshape value co-creation in smart urban tourism.

## 2 Literature review

### 2.1 Host–guest interaction in the context of tourism

Host–guest interaction is a classical topic in tourism studies and generally refers to the behavioral engagement, information exchange, and emotional responses that occur between tourists and local hosts in tourism settings [19]. Early studies mainly examined such interaction from the tourist perspective, showing that host–guest encounters significantly shape tourists’ destination perceptions, consumption decisions [20], and overall satisfaction [21]. In this sense, interaction was often treated as an experiential factor that enhances the quality of tourism encounters.

More recent scholarship has shifted from this relatively narrow view toward a broader understanding of host–guest interaction as a multi-actor and bidirectional process. On the one hand, increasing attention has been paid to the effects of interaction on residents’ attitudes, tourism support behaviors, and place identity. Frequent and positive interaction has been found to strengthen residents’ favorable perceptions of tourists and tourism development, while also enhancing community openness and tolerance toward tourism growth [22]. On the other hand, scholars have increasingly emphasized the role of local knowledge, cultural expression, and resident agency in shaping host–guest relations. For example, Zhang and Xu (2023) [23] argue that residents may undergo “role transformation” in tourism settings, thereby increasing their influence in local tourism discourse and interaction processes.

At the same time, the typology of both interaction subjects and interaction contexts has expanded considerably. Host–guest interaction is no longer limited to incidental face-to-face contact between tourists and individual residents; it increasingly involves a broader network of actors, including service personnel, business operators, and public institutions, thereby forming a more structured interactional system [24]. Moreover, interaction has evolved from one-way service delivery to multidimensional participation. It may take the form of affective interaction, such as informal communication and cultural exchange between tourists and residents [25], or institutional interaction, in which tourists engage in destination governance, information acquisition, and service feedback through smart platforms and public service systems [26].

This shift is especially important in the context of urban tourism in China, where local governments play a prominent intermediary role in tourism service provision, cultural resource integration, and smart-system development [27]. Under such conditions, host–guest interaction should not be regarded as a single homogeneous construct. Different host actors may offer different participation opportunities, resource access points, and relational meanings to tourists. Therefore, drawing on the practical characteristics of Chinese urban tourism and the expanding role of smart governance, this study conceptualizes host–guest interaction along two dimensions. The first is tourist–government interaction, which refers to institutional interaction through smart platforms, public services, and governance-related feedback mechanisms. The second is tourist–resident interaction, which refers to affective and culturally embedded interaction grounded in resident attitudes, local expression, and lifestyle contact. This distinction provides a more differentiated basis for examining how different interaction forms may shape tourists’ subsequent co-creation responses.

### 2.2 Value co-creation in the context of smart tourism

In recent years, value co-creation has become a central concept in smart tourism research, particularly as digital technologies have transformed the ways in which tourists engage with destinations, services, and local communities [28]. At its core, value co-creation challenges the traditional producer–consumer dichotomy by emphasizing that users actively participate in the creation of value through interaction, resource integration, and experience formation. In the tourism context, this means that tourists are no longer viewed as passive consumers of pre-designed services, but as active participants in cultural engagement, meaning-making, and destination value formation [29].

Theoretically, Service-Dominant Logic (SDL) provides an important foundation for understanding this process. SDL posits that service, rather than goods, is the fundamental basis of exchange, and that value is not delivered unilaterally but co-created through interaction and realized in use [30]. In tourism research, this perspective has been used to explain how tourists, residents, tourism enterprises, and digital platforms collectively contribute to value generation through ongoing interaction [31]. SDL is especially useful for the present study because it suggests that interaction matters not merely because it is socially pleasant, but because it enables actors to access, integrate, and mobilize resources within the destination service ecosystem.

From this perspective, tourism value co-creation can be understood as a multi-actor and multi-layered process. First, tourism enterprises increasingly stimulate tourist engagement through experience design, atmosphere creation, and participatory service encounters, thereby enhancing satisfaction and brand-related outcomes [32]. Second, peer-to-peer interaction among tourists has become an important co-creation mechanism, especially on social and sharing platforms where information exchange and emotional feedback generate new experiential fields [33]. Third, tourist–resident interaction has been shown to play a crucial role in local culture transmission, lifestyle recognition, and community participation, thereby shifting tourism value from mere consumption toward emotional connection and shared meaning [23].

In addition, residents are increasingly recognized as active participants rather than passive recipients of tourism development. Residents’ emotional attitudes toward tourists can shape the quality of visitor experiences and affect tourists’ willingness to engage in co-creation [34]. Their involvement in resource sharing, social interaction, cultural performance, ecological conservation, urban governance, and service design also makes them important nodes in the co-creation network [35, 36]. However, although existing studies have demonstrated that interaction is relevant to value co-creation, they have less often asked whether different forms of host–guest interaction provide different kinds of resources and participation conditions, and whether these interaction forms influence co-creation through the same or different psychological processes. This gap is particularly important in smart tourism settings, where interaction increasingly takes place across digital, institutional, and relational domains rather than within a single interpersonal context.

### 2.3 Artificial intelligence in the context of tourism

With the rapid integration of Artificial Intelligence (AI) into the tourism sector, AI technologies are increasingly reshaping service delivery, tourist participation, and the broader logic of host–guest interaction. As a result, AI has become a major focus of contemporary tourism research. Existing studies have primarily examined the technological benefits of AI in improving service efficiency, optimizing tourist experiences, and enhancing governance responsiveness. While these studies have established the practical importance of AI in tourism, they have paid less attention to how AI functions in different interactional settings and whether its effects on co-creation processes are context-dependent.

At the experiential level, AI technologies such as natural language processing, intelligent recommendation, emotion recognition, and virtual navigation can increase service responsiveness and information accessibility, thereby improving tourists’ perceptual efficiency and willingness to engage [37]. In personalized digital platforms, AI also enables real-time information delivery, customized service support, and more adaptive interaction strategies, which can strengthen experiential satisfaction and participation [38]. Recent findings further suggest that the successful integration of AI in tourism depends heavily on the depth of users’ engagement with intelligent systems, with higher engagement often leading to richer smart experiences and stronger affective responses [39]. Related work in e-commerce also indicates that different AI service forms significantly shape consumer adoption intentions [40].

However, these findings do not necessarily mean that AI exerts the same effect across all forms of tourism interaction. In host–guest interaction settings, the role of AI may vary depending on the nature of the interaction itself. AI may be more readily aligned with institutional and procedural forms of interaction that rely on information processing, service coordination, and feedback responsiveness. By contrast, its role may be more limited in affective and culturally embedded interactions that depend on emotional resonance, mutual recognition, and everyday social warmth. In other words, AI should not be treated simply as a universally beneficial technological tool; rather, it may function as a differentiated enabler whose influence depends on the social and institutional structure of the interaction context.

Therefore, identifying the boundary conditions and adaptation mechanisms of AI intervention in tourism interaction constitutes an important research task. In particular, current research has not sufficiently clarified whether AI strengthens the influence of different host–guest interaction types in the same way, or whether its moderating role is contingent on the actor relationship involved. By moving beyond the conventional assumption that AI uniformly enhances participation and experience, the present study examines AI technology stimuli as a context-sensitive boundary condition in the relationship between host–guest interaction and tourists’ co-creation responses. In doing so, it offers a more differentiated understanding of the AI–tourism nexus and of how intelligent technologies shape value co-creation in smart urban tourism.

### 2.4 Conceptual definitions of core constructs

Although the existing literature has discussed AI-related interaction, empowerment, and value co-creation from different perspectives, the terminology used across studies is not always consistent. To avoid conceptual ambiguity, this study adopts unified definitions for the three focal constructs in the proposed model: AI Technology Stimuli (AITS), Customer Empowerment (CE), and Co-creation Intention (CI). Their definitions, conceptual focus, and distinctions from related concepts are summarized in Table 1.

**Table 1:** Definitions of the core constructs.

Construct	Definition in this study	Conceptual focus	Distinction from related concepts
AI Technology Stimuli (AITS)	Tourists’ perceived AI-enabled cues and support signals embedded in destination platforms and smart service interfaces, such as intelligent guidance, real-time response, personalized recommendations, and feedback visibility.	Perceived AI-related interaction support	Differs from general technology adoption, system quality, and usability by focusing specifically on AI-related stimuli experienced during interaction processes.
Customer Empowerment (CE)	Tourists’ perceived capacity to express preferences, influence service-related processes, and participate meaningfully in destination value formation.	Perceived agency and influence in the destination service system	Differs from objective competence, actual decision-making power, general engagement, and satisfaction because it captures a psychological sense of being able to make a meaningful contribution.
Co-creation Intention (CI)	Tourists’ willingness and planned readiness to actively participate in activities that contribute to destination value creation, service improvement, or shared tourism experiences.	Behavioral intention toward co-creation	Differs from actual co-creation behavior, general participation, and positive attitude because it captures intended rather than realized action.

## 3 Method

### 3.1 Pilot site selection

The selection of the pilot site is primarily based on its distinctive urban tourism characteristics and the representativeness of host-guest interactions. As a renowned historical and cultural city and a national smart tourism demonstration city, Ningbo boasts abundant cultural tourism resources, including Tianyi Pavilion–Moon Lake Historical and Cultural District, Nantang Old Street, and Laowaitan. These locations not only cater to tourists’ sightseeing needs but also serve as vital spaces for local residents’ daily lives and cultural exchanges, making them ideal case studies for exploring tourist-resident interactions.

Moreover, Ningbo’s government leads the country in smart tourism development, leveraging digital tools such as the “Zheli Haowan” smart tourism platform and an integrated smart guidance system. These digital innovations facilitate multi-dimensional interactions between tourists and the local government, providing a solid empirical foundation for studying how smart technologies can enhance host-guest interactions [41].

Additionally, as a coastal port city with a high degree of commercial-tourism integration, Ningbo attracts a diverse range of visitors, making its host-guest interaction patterns highly representative. The research findings derived from this study not only offer insights for other tourism cities but also contribute to the development of a theoretical framework for host-guest interaction in the context of smart tourism.

Therefore, Ningbo’s diverse tourism scenarios and advanced smart tourism infrastructure provide an ideal empirical research environment, enhancing both the academic value and practical significance of this study.

### 3.2 Research approach

This study employed both qualitative and quantitative research methods. The research was conducted in two stages. First, prior studies on host–guest interaction, value co-creation, and artificial intelligence in tourism were reviewed, and grounded theory was used to refine the key constructs and relationships and support the development of the theoretical model. Second, quantitative analysis was conducted to test the proposed relationships and examine configurations associated with value co-creation intention. Partial least squares structural equation modeling was used to test direct, mediating, and moderating effects, while fuzzy-set qualitative comparative analysis was applied to identify alternative combinations of conditions associated with high and low value co-creation intention.All procedures were approved by the Ethics Committee of the Institute of Art and Technology Design, School of Design, NingboTech University (Approval No. NBT-ATD-EC-20241225).

### 3.3 Qualitative stage

In the qualitative stage, participants were tourists who had used the “Zheli Haowan” platform during their visit to Ningbo. Recruitment and interview data collection were conducted from 26 December 2024–20 January 2025. A total of 41 eligible adult tourists were recruited for focus group discussions and in-depth individual interviews. Before each interview, participants received an information sheet explaining the study purpose, voluntary participation, confidentiality protections, and the right to withdraw at any time. Written informed consent was obtained prior to data collection, and all interview materials were anonymized during transcription and analysis. The combination of focus group discussions and in-depth interviews enabled the study to capture both shared patterns of experience and more detailed individual reflections related to platform use, host–guest interaction, empowerment, and value co-creation in the smart tourism context. The interview data were analyzed using grounded theory procedures, including open coding, axial coding, and selective coding. Coding was conducted through iterative comparison across transcripts, and disagreements were resolved through discussion until a consensual coding scheme was reached. An audit trail, including coding memos and records of code refinement, was maintained to enhance analytic transparency and credibility..

### 3.4 Quantitative stage

In the quantitative stage, the target population consisted of tourists who had recent urban tourism experience in Ningbo and had been exposed to the “ZheliHaowan” platform or related smart tourism services. A non-probability sampling strategy was adopted. Survey recruitment and data collection were conducted from 6 February 2025–28 February 2025. The formal survey was administered primarily through Wenjuanxing (Questionnaire Star), with support from hotel industry associations and alumni networks in the Ningbo region to facilitate targeted recruitment of respondents with relevant tourism experience and platform exposure. A total of 560 questionnaires were collected. To ensure ethical compliance, respondents under 18 years of age were excluded from the sample. All questionnaires were completed anonymously, and no personally identifiable information was collected. After data cleaning, 507 valid questionnaires were retained for analysis. The quantitative analysis was conducted in two parts. First, partial least squares structural equation modeling was used to test the hypothesized relationships among tourist–government interaction, tourist–resident interaction, customer empowerment, artificial intelligence technology stimuli, and value co-creation intention, with particular attention to direct, mediating, and moderating effects. Second, fuzzy-set qualitative comparative analysis was applied to identify alternative combinations of interaction conditions, empowerment, and artificial intelligence technology stimuli associated with high and low levels of value co-creation intention.

### 3.5 Qualitative study on host-guest interaction types and their impacts‌‌

#### 3.5.1 Research procedure and data collection.

This qualitative study examines the characteristics and impact mechanisms of different host–guest interaction types in urban tourism. It is guided by two research questions: (1) How do tourists perceive, experience, and participate in host–guest interactions in urban tourism environments? (2) How do different types of host–guest interactions shape tourists’ travel experiences and subsequent behavioral responses?

To ensure the contextual relevance and content validity of the interview protocol, the research team developed an initial semi-structured guide based on the study objectives and prior literature, and then refined it through expert consultation. The expert panel included academic and practitioner stakeholders with experience in tourism management, destination governance, and tourism service operations, who provided feedback on question clarity, sensitivity, and coverage. The final protocol focused on four domains: (a) the process and situations of host–guest interaction during the trip, (b) tourists’ perceptions of interaction modes in urban tourism settings, (c) perceived impacts of interaction on travel experiences and emotions, and (d) behavioral responses under different interaction scenarios. The guide consisted of ten core questions, supplemented by follow-up probes to elicit concrete episodes and deepen interpretation (Table 2).

**Table 2:** List of intervew subject information.

No.	Gender	Age	Occupation	No.	Gender	Age	Occupation
1	M	28	Employee	22	M	20	Student
2	F	29	Employee	23	F	25	Student
3	F	41	Employee	24	M	38	Employee
4	M	46	Government Employee	25	F	38	Employee
5	M	19	Student	26	M	50	Government Employee
6	F	21	Student	27	M	33	Self-employed
7	F	36	Self-employed	28	F	42	Government Employee
8	F	42	Government Employee	29	F	55	Self-employed
9	F	40	Employee	30	F	19	Student
10	M	19	Student	31	M	21	Student
11	F	36	Self-employed	32	F	20	Student
12	M	22	Student	33	M	31	Self-employed
13	F	29	Employee	34	F	33	Self-employed
14	M	38	Self-employed	35	F	57	Government Employee
15	F	41	Government Employee	36	M	60	Retired
16	M	50	Employee	37	M	20	Student
17	M	65	Retired	38	F	24	Employee
18	M	57	Employee	39	M	30	Employee
19	F	45	Government Employee	40	F	29	Self-employed
20	F	41	Self-employed	41	F	26	Student
21	F	27	Employee				

Data were collected from tourists who had used the government-led smart tourism platform “ZheliHaoWan” during their visit to Ningbo. In line with a responsive interview approach [42], interviews were conducted in an open and dialogic manner to encourage participants to narrate their lived experiences in their own words, while the interviewer maintained a flexible probing strategy to clarify meanings, capture interaction details, and explore perceived consequences. Both focus group discussions and individual in-depth interviews were used to balance breadth and depth: focus groups helped surface shared perceptions and common interaction patterns, whereas individual interviews enabled more detailed accounts of personal experiences and sensitive reflections. To enhance procedural consistency, all interviews followed a standardized workflow: participants received an explanation of the study purpose and privacy protections, provided informed consent, completed basic demographic questions, and then proceeded to the interview. With participants’ permission, interviews were audio-recorded and later transcribed verbatim; identifying information was removed during transcription to protect confidentiality.

As analysis progressed, the interview guide was only minimally adjusted to improve probing and capture emergent themes, while keeping the core questions stable to maintain comparability across participants. Overall, the study collected approximately 610 minutes of recorded interviews and generated 142,000 words of transcripts, including 41,000 words from focus group discussions and 101,000 words from individual interviews. These materials provided a rich qualitative basis for subsequent grounded-theory coding and model development, with theoretical saturation and bias-control procedures reported in Section

#### 3.5.2 Grounded theory analysis.

This study adopted grounded theory as the main analytical approach for the qualitative stage for two reasons. First, grounded theory is particularly suitable for exploring micro-level, process-oriented social phenomena when existing theoretical explanations remain incomplete or insufficiently specific [43]. Given the contextual complexity and relational heterogeneity of host–guest interaction in urban tourism, this approach allowed the study to generate concepts inductively from tourists’ lived experiences rather than imposing a rigid pre-existing framework. Second, grounded theory emphasizes the iterative interplay between data collection, coding, comparison, and conceptual abstraction, making it appropriate for identifying how different interaction forms are perceived and linked to empowerment and value co-creation in smart tourism contexts [44].

To enhance procedural transparency and analytical rigor, all interview transcripts were imported into NVivo for data management and coding support. The software was used to organize transcripts, manage coding nodes, retrieve coded segments, compare emerging categories across interviews, and maintain links between coded excerpts and analytical memos. However, the coding decisions themselves were theory-driven and researcher-interpreted rather than mechanically generated by the software.

The coding process was conducted by two trained coders with prior experience in qualitative analysis. Before the formal coding began, both coders carefully read the transcripts several times to familiarize themselves with the interview content and to identify potentially relevant meaning units. A subset of transcripts was then selected for pilot coding, during which the two coders independently generated preliminary codes and compared their interpretations. Based on this process, an initial codebook was developed, including provisional code labels, brief definitions, inclusion criteria, and illustrative examples. The codebook was revised iteratively throughout the analysis to improve conceptual consistency and category clarity.

The formal analysis followed the three classic stages of grounded theory coding: open coding, axial coding, and selective coding. In the open coding stage, the transcripts were examined line by line, and meaningful expressions were coded using concise labels that captured actions, perceptions, and interactional experiences. Particular attention was paid to preserving the participants’ original meanings while reducing the raw narratives into analytically manageable conceptual units. Constant comparison was used throughout this stage to identify similarities and differences across cases, avoid redundant coding, and refine code boundaries.

In the axial coding stage, the initial codes were grouped and compared to identify conceptual relationships among categories. Codes that reflected similar conditions, interaction patterns, responses, or consequences were clustered together, and their internal logic was examined. This stage focused on clarifying how host–guest interaction forms, tourists’ psychological perceptions, contextual technology conditions, and co-creation-related outcomes were connected. Through repeated comparison and abstraction, broader subcategories and their relationships were progressively established.

In the selective coding stage, the analysis moved from category development to theoretical integration. The research team identified the core categories that best captured the central logic of the data and examined how these categories could be linked into a coherent explanatory framework. At this stage, only categories that were conceptually central and empirically well supported were retained in the final framework. This process enabled the study to move beyond descriptive classification and construct a grounded explanation of how different host–guest interaction types relate to empowerment and value co-creation intention in smart tourism settings.

To strengthen coding reliability, the two coders independently coded the same subset of transcripts at multiple points during the analysis and then compared their coding results. Discrepancies were discussed in detail, and disagreements were resolved through iterative discussion with reference to the transcript context and the evolving codebook. When necessary, the senior author reviewed disputed segments and provided adjudication to ensure conceptual consistency. In parallel, memo-writing was conducted throughout the entire process to document emerging insights, category revisions, coding uncertainties, and theoretical reflections. The revision history of the codebook, together with the coding memos and records of coder discussions, formed an audit trail that enhanced the traceability and transparency of the analytical procedure.

Through this iterative process, the qualitative analysis generated a progressively refined conceptual structure that served as the basis for subsequent model development in the quantitative stage.

(1) **Open Coding**

Based on the original interview data, this study extracted preliminary codes and iteratively compared and restructured concepts to identify core theoretical constructs that explain the research problem [45]. During the open coding phase, a line-by-line coding approach was employed to ensure the comprehensive capture of implicit information in the raw data, minimizing potential omissions. Additionally, a hierarchical coding strategy was implemented to maintain a close connection between initial codes and the original data, enhancing the systematicity and accuracy of data interpretation.

In the categorization process, particular attention was given to the attributes and internal hierarchy of different concepts. The study systematically examined the logical relationships among multiple concepts within the same category, gradually refining their levels of abstraction. Based on this structured approach, a substantive theoretical framework closely aligned with the research problem was developed. Table 3 provides examples illustrating the transformation from raw data to conceptual formation.Through systematic coding and conceptual synthesis, this study ultimately extracted 389 initial codes, which were further consolidated into 33 core concepts, providing a robust data foundation for subsequent theoretical development.

**Table 3:** Example of the formation process from raw materials to concepts (partial).

Original Data Excerpt	Initial Concept	Category
a1: I often ask local residents for directions, such as how to get to the historical district, and they always provide detailed guidance. a2: I inquire about how local traditional festivals are celebrated, and residents are always happy to share their experiences with me. a3: I have participated in community-organized activities, such as joining a community garden workshop with local residents, which was very meaningful. a4: I stay at a guesthouse and often engage in conversations with the owner about local culture and customs.	a1: Residents provide information to tourists. a2: Tourists share local culture or traditional customs with residents. a3: Tourists participate in community activities. a4: Residents host tourists through guesthouses, establishing communication with them.	A1: Tourist and resident interaction
a5: I often use the guided tours provided by the government to learn about the scenic area, such as recommended routes and important tips. a6: The smart tourism platform is especially convenient, allowing me to book tickets and reserve guided tours in advance, saving a lot of time. a7: The local government frequently organizes cultural promotional activities, such as intangible cultural heritage exhibitions, which help me gain a deeper understanding of the local culture. a8: This year, the government introduced a cultural festival discount policy, and we took the opportunity to visit several exhibitions, which were very rewarding. a9: When participating in certain activities, we have the right to offer suggestions, such as choosing the way and content of interaction, which gives me a strong sense of accomplishment.	a5: Tourists obtain travel information through government-guided tours. a6: The smart tourism platform provides convenient service booking functions. a7: The government organizes cultural promotional activities to enhance interactive experiences. a8: The cultural discount policies introduced by the government attract tourists to participate. a9: Tourists provide feedback and suggestions through the smart platform.	A2: Tourist and government interaction
a10: After participating in the community sharing activities, I gained a deeper understanding of the village’s culture and learned many new and interesting things. a11: Communicating with community members helped me better understand their needs and interests, and it also allowed me to rediscover the unique charm of the community. a12: I noticed that community members were very actively engaged in our activities, which gave me confidence in their local culture and increased my anticipation for future development. a13: During the community activities, I had the opportunity to vote on the topics I was most interested in, which made me feel truly involved and gave me a great sense of accomplishment. a14: As a guide during the activity, interacting with other tourists was very meaningful, and the entire experience was quite unique. a15: At the creative market, I not only had the chance to present my own ideas but also collaborated with local residents to create a cultural exhibition plan, which made me feel deeply connected to the local culture.	a10: Tourists gain more decision-making and participation rights through interaction. a11: Tourists strengthen their emotional connection to the destination through activities. a12: The interaction process enhances mutual cultural respect. a13: Tourists gain decision-making power and influence the design of activities by participating in local event planning. a14: Tourists improve their understanding and respect for local traditions through interactive educational activities. a15: Tourists provide suggestions in community creative renewal projects, contributing to co-creation plans.	A3: customer empowerment

(2) **Axial Coding**

Axial coding aims to identify the relationships between initial categories and further clarify their underlying meanings [45]. In this stage, the study systematically organized and analyzed the open coding results using inductive analysis, establishing theoretical connections among core categories. Through repeated comparison and iteration, 33 concepts were consolidated and integrated into 8 subcategories, enhancing the clarity of the data structure and the coherence of the theoretical framework. (Table 4).

**Table 4:** Comparison table between subcategories and initial concepts.

Subcategory	Initial Concept
A1:Tourist and resident interaction	a1: Residents provide information to tourists, such as directions or recommendations for attractions. a2: Tourists share cultural stories or traditional customs with residents. a3: Tourists participate in community activities. a4: Residents host tourists through guesthouses, establishing communication with them.
A2:Tourist and government interaction	a5: Tourists obtain tourism information through government-guided services. a6: The smart tourism platform provides convenient service booking functions. a7: The government organizes cultural promotional activities to enhance the interactive experience. a8: Cultural discount policies introduced by the government attract tourists to participate. a9: Tourists provide feedback and suggestions through the smart platform.
A3:customer empowerment	a10: Tourists gain more decision-making and participation rights during interactions. a11: Tourists strengthen their emotional connection to the destination through activities. a12: The interaction process enhances mutual cultural respect. a13: Tourists gain decision-making power and influence activities by participating in local event planning. a14: Residents improve tourists’ understanding and respect for local traditions through interactive educational activities. a15: Tourists provide suggestions in community creative renewal projects, contributing to co-creation plans.
A4:Interaction outcomes	a16: Tourists co-create local handicrafts with residents. a17: Tourists participate in cultural heritage activities, promoting the cultural transmission of the estination. a18: Residents and tourists collaborate to develop tourism routes, optimizing the experience. a19: Tourists participate in local folk festivals and celebrations.
A5:Artificial intelligence stimuli	a20: AR technology enhances tourists’ understanding of historical and cultural contexts. a21: Smart voice assistants provide real-time translation services, improving interaction. a22: Entertainment information push in smart systems promotes tourists’ willingness to engage in experiences. a23: Smart guided devices increase the enjoyment of interactions during the experience.
A6:Cultural identity	a24: Historical districts become primary locations for communication between tourists and residents. a25: Iconic architecture and cultural heritage enhance the destination’s appeal. a26: Open spaces, such as city squares, provide physical platforms for interaction.
A7: Collaborative ability	a27: Residents and tourists collaborate on community projects, such as environmental actions. a28: Tourists and residents co-participate in community creative activities. a29: Tourists and residents collaborate to plan cultural exhibition activities.
A8:Social networks	a30: Interactions promote tourists’ cross-cultural understanding and network expansion. a31: Social networks help tourists access more information about the destination. a32: Online community platforms drive the diverse development of interactions. a33: Social media facilitates two-way feedback communication between residents and tourists.

(3) **Selective Coding**

During the selective coding phase, this study focused on analyzing the theoretical relationships among the main categories and identifying the core category based on the structured relationships [45]. Through an abstract synthesis of axial coding results, five main categories were derived: host-guest interaction types, psychological cognition of interaction, interaction outcomes, contextual factors, and value co-creation drivers triggered by host-guest interaction. A detailed comparison with the original interview data revealed the mechanistic relationships between the five main categories and the eight subcategories (Table 5). Based on these findings, a typical model mechanism diagram was constructed to illustrate the theoretical framework.

**Table 5:** Category relationship and category connotation between main and subcategories.

Main Category	Subcategory	Category Concept
B1: Host-guest interaction forms	A1:Tourist and resident interaction	Host-guest interactions between tourists and residents in cultural exchanges, daily conversations, and experiential activities promote emotional connections and cultural dissemination.
	A2:Tourist and government interaction	Tourists interact with local governments through smart tourism platforms, policy services, or city tours, enhancing service efficiency and the overall tourism experience.
B2: Psychological cognition of interaction	A3:customer empowerment	Through host-guest interactions, tourists are granted greater autonomy in participation, enhancing their sense of role and engagement.
B3: Interaction outcomes	A4:Interaction outcomes	Interaction promotes value co-creation among tourists, residents, and the destination, achieving mutual benefits for all parties.
B4: Contextual factors	A5:Artificial intelligence stimuli	Smart devices provide stimulation and convenience for interactions, optimizing the overall experience.
B5: Value co-creation drivers triggered by host-guest interaction	A6:Cultural identity	During interactions, tourists develop a sense of cultural identity with the destination.
	A7: Collaborative ability	The interaction process fosters the enhancement of collaboration skills between tourists and residents.
	A8:Social networks	Through interaction, tourists expand their social networks, establish closer ties with residents, and increase overall satisfaction with the experience.

#### 3.5.3 Theoretical saturation and bias control.

To ensure the credibility and trustworthiness of the grounded theory analysis, this study adopted multiple strategies to mitigate potential biases and confirm theoretical saturation. First, open coding was conducted using a line-by-line approach, supported by constant comparison and reflective memo-writing, to minimize subjectivity and enhance analytical rigor. Second, two trained coders independently coded the transcripts. Coding disagreements were discussed and, when necessary, adjudicated by the senior author based on textual evidence. A codebook was iteratively refined across coding rounds, and the revision history and analytic memos were archived as part of the audit trail to enhance transparency and reliability. Third, the full dataset comprising interviews with 41 participants was divided into two groups: 30 interviews were used to conduct the three-stage coding process (open, axial, and selective coding) and construct the initial theoretical framework, while the remaining 11 interviews were reserved for saturation testing. After completing the coding of the initial 30 interviews and developing a conceptual model, the reserved 11 interviews were then analyzed. No new concepts or categories emerged during this phase, indicating that the theoretical framework adequately captured the core patterns in the data. Thus, theoretical saturation was considered achieved, and the risk of bias from over-reliance on a limited sample was effectively controlled.

#### 3.5.4 Qualitative findings.

**Fig 1:**
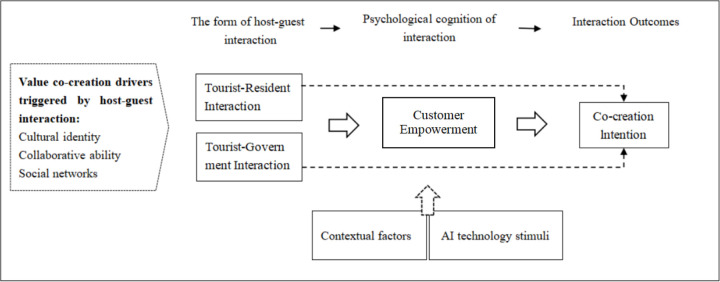
Theeoretical relationships of the co-creation willingness impact mechanism based on host-guest interaction types.

As shown in Fig 1, the qualitative analysis identified several categories related to value co-creation in host–guest interaction, including interaction forms, empowerment-related perceptions, contextual technology conditions, and value co-creation drivers. Among these, tourist–resident interaction and tourist–government interaction emerged as two distinct forms of host–guest interaction in the smart urban tourism context.

The interview data indicated that tourist–resident interaction was more frequently associated with emotional connection, local cultural contact, and participation in community-based experiences. By contrast, tourist–government interaction was more often linked to service support, information access, smart guidance, and platform-based engagement. Although these two interaction forms differed in their experiential emphasis, both were associated with tourists’ perceived empowerment, particularly in terms of autonomy, participation, and expression opportunities [46]. Participants described greater autonomy, stronger participation awareness, and more opportunities to express opinions or provide feedback through both resident-based and government-supported interactions.

In addition, smart tourism technologies and related contextual conditions were repeatedly mentioned by participants as factors shaping the interaction experience. Artificial intelligence-related functions, such as smart guidance, service recommendations, and digital feedback channels, appeared particularly salient in tourist–government interaction. Overall, the qualitative findings suggest that different host–guest interaction types may be associated with value co-creation intention through empowerment-related perceptions, while technology conditions may influence these relationships in different ways [47].

### 3.6 Hypotheses and model

#### 3.6.1 Host–guest interaction and co-creation intention.

Drawing on social exchange theory, host–guest interaction can be understood as a reciprocal exchange process in which tourists and hosts evaluate the benefits and meaningfulness of engagement during tourism experiences [18]. In this study, host–guest interaction refers to the exchanges between tourists and two key host actors in smart urban tourism contexts, namely local residents and government authorities. Such interaction may provide tourists with information, emotional support, participation opportunities, and feedback channels, thereby increasing their willingness to engage beyond passive consumption.

Prior studies suggest that host–guest interaction can promote value co-creation by providing both relational and instrumental resources [48]. Tourist–resident interaction is mainly rooted in interpersonal communication and cultural contact. Through conversations, shared activities, and local cultural exchange, tourists may develop stronger emotional connection, trust, and willingness to participate in destination-related activities, which can enhance co-creation intention [49,50]. Tourist–government interaction is more closely linked to institutional support and service coordination. When governments provide responsive services, participation mechanisms, and feedback channels through smart tourism systems, tourists are more likely to perceive their participation as useful and worthwhile, thereby strengthening their co-creation intention [51, 52].

Therefore, both tourist–resident interaction and tourist–government interaction are expected to positively influence tourists’ co-creation intention in smart urban tourism contexts. Accordingly, this study proposes the following hypotheses:

H1a: Tourist–Government Interaction (TGI) has a positive impact on Co-creation Intention (CI).

H1b: Tourist–Resident Interaction (TRI) has a positive impact on Co-creation Intention (CI).

#### 3.6.2 The mediating role of customer empowerment (CE).

Customer Empowerment (CE) refers to a psychological state in which tourists feel that they have sufficient information, autonomy, and influence to participate effectively in tourism experiences and service processes [12,53]. In the context of smart urban tourism, empowerment is important because value co-creation requires tourists to move from passive service users to active participants. When tourists feel that their choices matter, that they can contribute meaningfully, and that their participation can make a difference, they are more likely to engage in co-creation activities [54].

Host–guest interaction can enhance tourists’ empowerment by providing information, support, recognition, and opportunities for participation [55,56]. Through interaction with residents, tourists may gain local knowledge, social acceptance, and greater confidence in participating in cultural and community-based activities. Through interaction with government-related service systems, tourists may experience clearer procedures, more responsive feedback, and stronger perceptions that their opinions can influence tourism services and destination management. In both cases, interaction can strengthen tourists’ sense of autonomy, competence, and participation, thereby increasing customer empowerment [9, 57–59].

Customer empowerment is further expected to promote co-creation intention. When tourists feel empowered, they are more likely to provide suggestions, share ideas, and participate in collaborative activities because they perceive their involvement as effective and worthwhile [60,61]. Therefore, customer empowerment is expected to function as a key psychological mechanism linking host–guest interaction to co-creation intention.

Accordingly, this study proposes the following hypotheses:

H2a: Tourist–Resident Interaction (TRI) has a positive impact on Customer Empowerment (CE).

H2b: Tourist–Government Interaction (TGI) has a positive impact on Customer Empowerment (CE).

H3a: Customer Empowerment (CE) mediates the relationship between Tourist–Government Interaction (TGI) and Co-creation Intention (CI).

H3b: Customer Empowerment (CE) mediates the relationship between Tourist–Resident Interaction (TRI) and Co-creation Intention (CI).

#### 3.6.3 The moderating role of AI technology stimuli.

Artificial intelligence is increasingly embedded in smart tourism platforms and can influence how tourists interact with residents, service systems, and destination management processes [62]. In this study, AI technology stimuli refer to tourists’ perceived platform-based stimuli generated by AI-enabled functions during destination engagement. These stimuli may enhance interaction by making communication, information access, feedback, and participation more efficient, transparent, and engaging [63].

AI technology stimuli are expected to strengthen the effects of host–guest interaction because they reduce friction in the interaction process and increase tourists’ sense of control and responsiveness. In tourist–government interaction, AI-enabled systems such as real-time service response, smart guidance, and feedback tracking can help tourists access information more easily, understand procedures more clearly, and perceive that their participation is being processed and responded to. Under stronger AI technology stimuli, tourist–government interaction is therefore more likely to enhance customer empowerment and co-creation intention [64–66].

A similar strengthening effect may also occur in tourist–resident interaction. AI-enabled functions such as translation support, recommendation matching, and participation guidance can facilitate communication and coordination between tourists and residents, making interaction more accessible and manageable [67]. When these technology stimuli are stronger, tourists may feel more capable of engaging with residents, participating in local experiences, and translating interaction into meaningful contribution. As a result, tourist–resident interaction is more likely to strengthen customer empowerment and co-creation intention.

Accordingly, this study proposes the following hypotheses:

H4a: AI Technology Stimuli (AITS) positively moderate the relationship between Tourist–Government Interaction (TGI) and Customer Empowerment (CE), such that the relationship is stronger when AITS are higher.

H4b: AI Technology Stimuli (AITS) positively moderate the relationship between Tourist–Resident Interaction (TRI) and Customer Empowerment (CE), such that the relationship is stronger when AITS are higher.

H5a: AI Technology Stimuli (AITS) positively moderate the relationship between Tourist–Government Interaction (TGI) and Co-creation Intention (CI), such that the relationship is stronger when AITS are higher.

H5b: AI Technology Stimuli (AITS) positively moderate the relationship between Tourist–Resident Interaction (TRI) and Co-creation Intention (CI), such that the relationship is stronger when AITS are higher.

**Fig 2:**
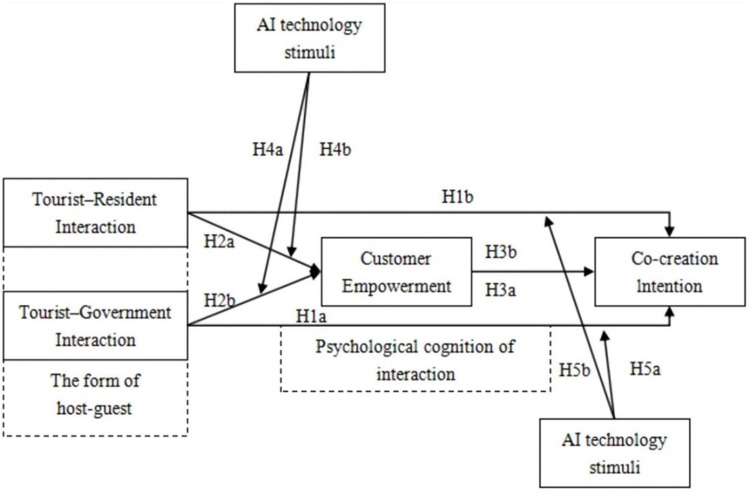
Model.

Based on the findings from the qualitative study and the theoretical reasoning derived from existing literature, this study constructs the research model as illustrated in Fig 2.

## 4 Verification of the path mechanism between host-guest interaction types and tourists’ co-creation intention

### 4.1 Survey design

Based on the theoretical model, this study collects data through a survey, which is divided into two sections: demographic information (age, gender, occupation, education level, income) and measurement items related to the research model. All research variables (excluding demographic information) are evaluated using a 7-point Likert scale (Table 6).

**Table 6:** Summary of the construct items.

Initial dimension	Code and Item description
Tourist-Government Interaction (TGI)	TGI1: Tourism information provided by the municipal government is timely.
	TGI2: Communication channels provided by the municipal government are effective.
	TGI3: The municipal government interacts with me in a friendly manner.
	TGI4: The municipal government responds positively to my feedback.
Tourist-Resident Interaction (TRI)	TRI1: It is easy to communicate with local residents.
	TRI2: Local residents are willing to answer my questions.
	TRI3: I can build friendships with local residents.
	TRI4: Local residents are willing to share their experiences of using the smart tourism platform with me.
Co-creation lntention (CI)	CI1: I find value co-creation through the smart tourism platform interesting.
	CI2: I am willing to use the smart tourism platform for value co-creation.
	CI3: I am willing to offer more help to improve the smart tourism platform.
	CI4: I think value co-creation through the smart tourism platform is a good idea.
Customer Empowerment (CE)	CE1: When interacting with this service provider, I feel in control of the situation.
	CE2: Being able to influence the goods and services provided by this service provider benefits me.
	CE3: I feel pleased that I can influence the options offered to me by this service provider.
	CE4: Compared with past travel experiences, I feel my influence over this service provider has increased.
AI technology stimuli (AITS)	
Passion (P)	P1: I am willing to use the smart tourism platform because I feel enthusiastic about it.
	P2: I enjoy using the smart tourism platform because I can use my experience to help others.
	P3: I enjoy helping other users by answering their questions on the smart tourism platform.
	P4: I feel pleased when I can help answer other users’ questions on the smart tourism platform.
Usability (U)	U1: Everything is easy to understand when using the smart tourism platform.
	U2: I can easily obtain the information I need when using the smart tourism platform.
	U3: I feel in control of what I can do when using the smart tourism platform.
	U4: I receive fast service when using the smart tourism platform.

The measurement items for host–guest interaction were adapted from prior studies [68–70]. Tourists’ co-creation intention was assessed using four items adapted from Tao et al. (2022) [71] and Lan et al. (2021) [72]. Customer empowerment was measured using four items drawn from Xie et al. (2020) [12] and Mohammad (2020) [73]. For AI technology stimuli, the passion dimension was adapted from Cao and Liu (2023) [74], whereas the usability dimension was adapted from the Chatbot Usability Scale developed by Borsci et al. (2022) [75].

Prior to the official survey, a pilot study was conducted, distributing 50 pre-test questionnaires, with 42 valid responses received. The validity of the scales was confirmed through Cronbach’s alpha values, all of which exceeded the recommended threshold of 0.70, ensuring reliability and internal consistency.

### 4.2 Sample characteristics

According to the frequency analysis results, 52.86% of the respondents are male (268 individuals), and 47.14% are female (239 individuals). In terms of occupation, government employees and self-employed individuals represent 26.23% (133 individuals) and 26.43% (134 individuals), respectively, while students and corporate employees account for 22.88% (116 individuals) and 24.46% (124 individuals). Regarding educational background, the largest group holds a bachelor’s degree, representing 34.71% (176 individuals), followed by high school or lower (32.94%, 167 individuals) and master’s degree or higher (32.35%, 164 individuals). In terms of age distribution, 17.55% (89 individuals) are aged 18 or younger, 20.32% (103 individuals) are between 18–25 years, 16.57% (84 individuals) are between 26–35 years, 16.57% (84 individuals) are between 36–45 years, 16.17% (82 individuals) are between 46–55 years, and 12.82% (65 individuals) are aged 56 or above. Regarding income, 23.47% (119 individuals) earn 2999Yuan or less, 25.64% (130 individuals) earn between3000–5999Yuan,24.85%(126 individuals) earn between 6000–8999Yuan, and 26.04% (132 individuals) earn 9000Yuan or more.The demographic characteristics of the sample are detailed in Table 7.

**Table 7:** Sample characteristics of the respondents.

Category	Option	Frequency	%
Gender	Male	268	52.860
	Female	239	47.140
Occupation	Student	116	22.880
	Government Employee	133	26.233
	corporate Employee	124	24.458
	self-employed	134	26.430
EducationLevel	High School or Below	167	32.939
	Bachelor’s Degree	176	34.714
	Master’s Degree or Higher	164	32.347
Age	=18	89	17.554
	18-25	103	20.316
	26-35	84	16.568
	36-45	84	16.568
	46-55	82	16.174
	≥56	65	12.821
Income	≤2999**¥**	119	23.471
	3000-5999**¥**	130	25.641
	6000-8999**¥**	126	24.852
	≥9000**¥**	132	26.036
Total		507	100.0

### 4.3 Data analysis and hypothesis testing

The quantitative analysis was conducted using Smart PLS 3.0 software for Partial Least Squares Structural Equation Modeling (PLS-SEM) to test all hypotheses [76]. Currently, PLS-SEM is widely applied in research within the hospitality field. This method does not require data to follow a strict normal distribution, making it suitable for analyzing formative models, complex models, and higher-order models.

### 4.4 Measurement model fit

To further assess the adequacy of the measurement model, a confirmatory factor analysis (CFA) was conducted. The results indicated a good model fit to the data (χ²/df = 1.3662, CFI = 0.9834, TLI = 0.9807, RMSEA = 0.0269), with all indices meeting the recommended thresholds.(Table 8)

**Table 8:** Measurement model fit indices.

Fit Index	Value
Chi-square (χ²)	323.7812
Degrees of freedom (df)	237
χ²/df	1.3662
CFI	0.9834
TLI	0.9807
RMSEA	0.0269

### 4.5 Reliability and convergent validity

In this study, Cronbach’s alpha values for all latent variables exceeded 0.80, indicating satisfactory internal consistency reliability. In addition, the standardized factor loadings of all observed variables were above 0.70. The Average Variance Extracted (AVE) values for all constructs were greater than 0.50, and the Composite Reliability (CR) values were above 0.70, indicating satisfactory convergent validity of the measurement scales (Table 9).

**Table 9:** Cronbach’s α, AVE and CR values.

Latent Variable	Observed Variable	Factor Loading	Cronbach’s α	AVE	CR
CE	CE1	0.857	0.85	0.69	0.899
	CE2	0.814			
	CE3	0.826			
	CE4	0.826			
CI	CI1	0.825	0.841	0.675	0.893
	CI2	0.814			
	CI3	0.832			
	CI4	0.816			
P	P1	0.831	0.811	0.638	0.876
	P2	0.791			
	P3	0.798			
	P4	0.774			
TGI	TGI1	0.869	0.874	0.726	0.914
	TGI2	0.851			
	TGI3	0.841			
	TGI4	0.846			
TRI	TRI1	0.849	0.861	0.706	0.906
	TRI2	0.839			
	TRI3	0.842			
	TRI4	0.832			
U	U1	0.821	0.81	0.637	0.875
	U2	0.785			
	U3	0.793			
	U4	0.794			

### 4.6 Discriminant validity

As shown in Table 8, all HTMT values among the constructs were below the threshold of 0.85, indicating satisfactory discriminant validity.(Table 10)

**Table 10:** HTMT values.

	CE	CI	P	TGI	TRI	U
CE						
CI	0.53					
P	0.065	0.071				
TGI	0.478	0.246	0.068			
TRI	0.493	0.278	0.076	0.417		
U	0.045	0.138	0.339	0.045	0.033	

### 4.7 Common method bias

This study used Harman’s single-factor test to assess potential common method bias [77]. As shown in Table 9, the variance explained by the first unrotated factor was 24.15%, which is below the commonly accepted threshold of 40%, indicating that common method bias is unlikely to be a serious concern in this study.(Table 11)

**Table 11:** Results of Harman’s single-factor test.

Factor	Variance Explained (%)	Cumulative Variance Explained (%)
Factor 1	24.15	24.15
Factor 2	13.77	37.92
Factor 3	9.84	47.76
Factor 4	7.83	55.59
Factor 5	7.47	63.05
Factor 6	5.42	68.47

### 4.8 Structural model evaluation

**Fig 3:**
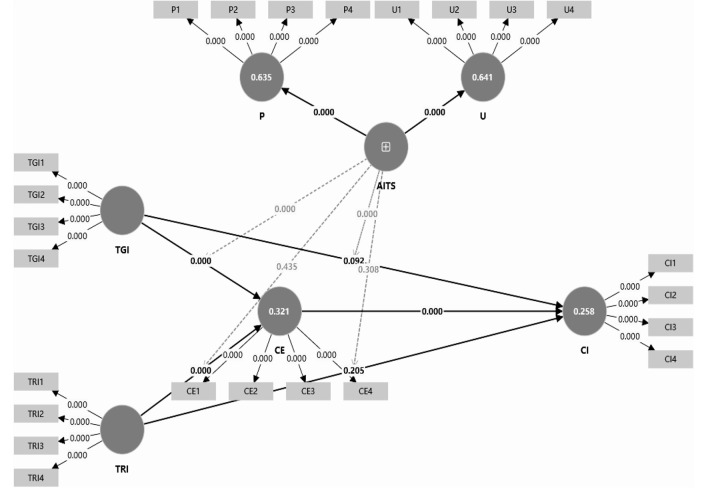
Structural equation modeling analysis results.

This study used SmartPLS for structural model evaluation. The specific model construction and results are shown in Fig 3.

#### 4.8.1 Multicollinearity test.

This study used the Variance Inflation Factor (VIF) to test for multicollinearity among the research variables. The results show that the VIF values for all research variables are below 5, indicating that there is no multicollinearity issue in this study.(Table 12)

**Table 12:** Multicollinearity test.

	VIF
AITS - > CE	1.002
AITS - > CI	1.009
AITS - > P	1
AITS - > U	1
CE - > CI	1.472
TGI - > CE	1.239
TGI - > CI	1.41
TRI - > CE	1.196
TRI - > CI	1.303
AITS x TRI - > CE	1.313
AITS x TRI - > CI	1.313
AITS x TGI - > CE	1.338
AITS x TGI - > CI	1.427

#### 4.8.2 R^2^ and f^2^.

The model demonstrates moderate explanatory power for both endogenous constructs. Specifically, host–guest interactions, AI-related conditions, and their interaction terms explain 32.1% of the variance in customer empowerment (CE; R^2^ = 0.321) and 25.8% of the variance in co-creation intention (CI; R^2^ = 0.258) (Table 13). Given that CI is a high-level motivational intention shaped by multiple unobserved situational and personal factors in real tourism settings, this level of explained variance is consistent with prior behavioral research and supports the model’s utility as a mechanism-diagnostic framework rather than a purely predictive tool [78]. Effect-size estimates further show that several focal direct paths to CI (e.g., TGI → CI; TRI → CI) and some AI-related terms exhibit small incremental contributions (f^2^ < 0.02) (Table 14). Importantly, these small f^2^ values do not undermine the model’s theoretical payoff: the results indicate that co-creation intention is not primarily driven by interaction “per se,” but by whether interaction is translated into an empowerment experience, and whether AI functions as a context-specific amplifier in institutional interaction settings.

**Table 13:** R^2^.

	R Difference	Adjusted R²
CE	0.321	0.314
CI	0.258	0.249

**Table 14:** f^2^.

	f²
AITS - > CE	0.007
AITS - > CI	0.013
AITS - > P	1.742
AITS - > U	1.785
CE - > CI	0.121
TGI - > CE	0.138
TGI - > CI	0.004
TRI - > CE	0.089
TRI - > CI	0.001
AITS x TRI - > CE	0
AITS x TRI - > CI	0
AITS x TGI - > CE	0.066
AITS x TGI - > CI	0.037

#### 4.8.3 Path analysis.

This study used SmartPLS software to test the path effects, with the results including standardized coefficients, t-values, and p-values, as shown below:(Table 15)

**Table 15:** Path analysis.

	β	Se	t	p
CE - > CI	0.363	0.044	8.166	0
TGI - > CE	0.34	0.039	8.685	0
TGI - > CI	0.061	0.046	1.328	0.092
TRI - > CE	0.269	0.04	6.801	0
TRI - > CI	0.038	0.046	0.825	0.205

The path analysis results suggest that host–guest interaction does not directly translate into co-creation intention in the smart tourism platform context. Specifically, Customer Empowerment (CE) exerts a significant positive effect on Co-creation Intention (CI) (β = 0.363, p < 0.001). Both Tourist–Government Interaction (TGI) and Tourist–Resident Interaction (TRI) significantly predict CE (TGI → CE: β = 0.340, p < 0.001; TRI → CE: β = 0.269, p < 0.001), whereas their direct effects on CI are not significant (TGI → CI: β = 0.061, p = 0.092; TRI → CI: β = 0.038, p = 0.205) (Table 12). Institutionally, platform-mediated government interactions are often procedural and bounded by standardized service routines, which may be perceived as “service handling” rather than participation; psychologically, resident interactions may generate positive feelings but remain low-stakes if tourists do not perceive real influence on service design or experience shaping. Therefore, interaction alone is not a sufficient net-effect driver of CI; its behavioral relevance depends on whether tourists interpret interaction experiences as expanding their agency and influence in the destination service system [79].

#### 4.8.4 Mediation effects.

This study used SmartPLS software to test the mediation effects, as shown below:(Table 16)

**Table 16:** Mediation effect analysis.

	Effect Value	Se	t	p	5.00%	95.00%
TGI - > CE - > CI	0.124	0.021	5.776	0	0.089	0.16
TRI - > CE - > CI	0.098	0.02	4.932	0	0.066	0.131

To examine whether empowerment functions as the psychological conduit linking interaction to co-creation intention, mediation effects were assessed using bootstrapping. The indirect effect of TGI on CI via CE is significant (TGI → CE → CI = 0.124; t = 5.776, p < 0.001), and the 90% percentile bootstrap interval (5th–95th) excludes zero [0.089, 0.160], supporting H3a (Table 13). Likewise, the indirect effect of TRI on CI via CE is significant (TRI → CE → CI = 0.098; t = 4.932, p < 0.001), with a bootstrap interval excluding zero [0.066, 0.131], supporting H3b. Given that the corresponding direct effects are non-significant (TGI → CI: p = 0.092; TRI → CI: p = 0.205), the mediation pattern is best characterized as indirect-only (full mediation). This implies that host–guest interaction shapes co-creation intention primarily by generating an empowerment experience (e.g., perceived voice, control, and efficacy), rather than exerting a direct motivational push on CI [80].

#### 4.8.5 Moderating effect test.

**Fig 4:**
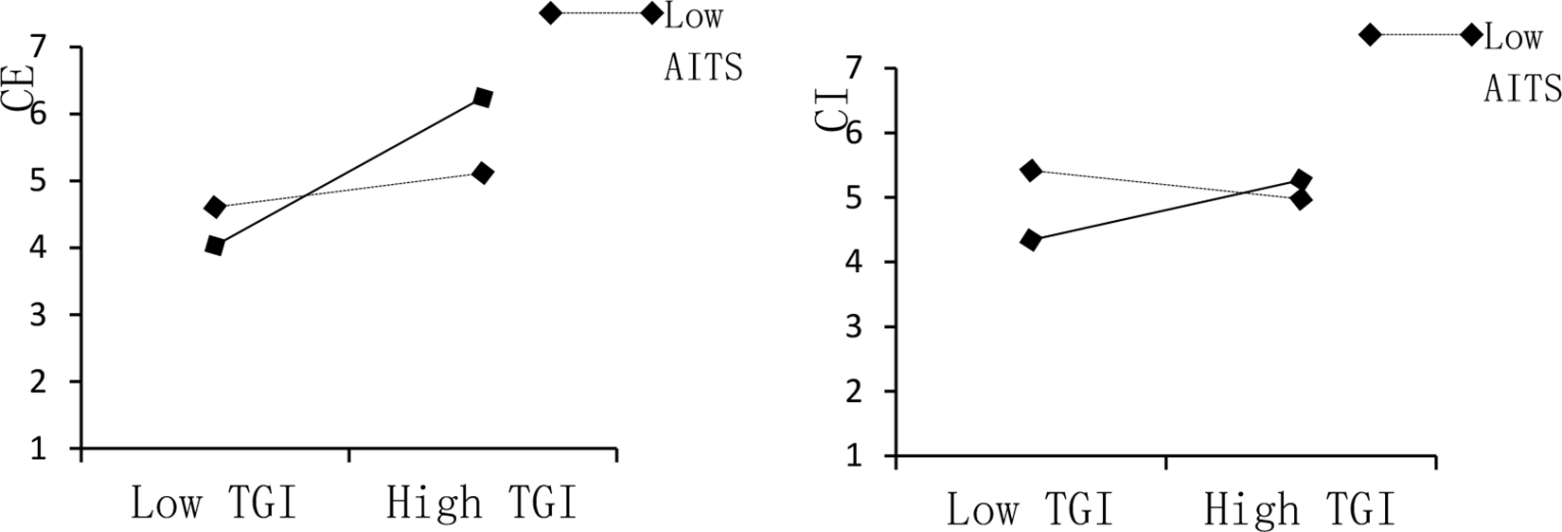
Moderating effect.

Moderation tests further reveal that the boundary role of AI technology stimuli (AITS) is context-dependent. In the tourist–resident interaction context, the interaction terms are non-significant for both AITS × TRI → CE (β = 0.007, p = 0.435) and AITS × TRI → CI (β = 0.021, p = 0.308), providing no support for H4b and H5b (Table 14). In contrast, AITS significantly strengthens the effects of tourist–government interaction: AITS × TGI positively predicts CE (β = 0.213, p < 0.001), supporting H4a, and also positively predicts CI (β = 0.171, p < 0.001), supporting H5a. This pattern suggests that AI-enabled stimuli primarily operate as an institutional-interaction amplifier—enhancing the perceived feasibility and consequentiality of participation in governance-related channels—rather than functioning as a universal enhancer of all host–guest interactions [81]. As illustrated in Table 17 and Fig 4, the positive influence of TGI on both CE and CI becomes stronger when AITS is higher, indicating clear contextual heterogeneity in the role of AI in facilitating co-creation processes.

**Table 17:** Moderating effect test.

	β	Se	t	p
AITS x TRI - > CE	0.007	0.04	0.165	0.435
AITS x TRI - > CI	0.021	0.042	0.5	0.308
AITS x TGI - > CE	0.213	0.035	6.168	0
AITS x TGI - > CI	0.171	0.04	4.289	0

## 5 Fuzzy-set qualitative comparative analysis (fsQCA)

This study first employed partial least squares structural equation modeling (PLS-SEM) to examine the hypothesized net effects and indirect mechanisms among Tourist–Government Interaction (TGI), Tourist–Resident Interaction (TRI), Customer Empowerment (CE), and Co-creation Intention (CI), as well as the moderating role of AI-enabled platform stimuli (AITS). This correlation-based approach enables the assessment of linear relationships, path coefficients, and mediation/moderation effects within the proposed theoretical model.

However, value co-creation in smart urban tourism is likely driven by causal complexity, where multiple conditions may operate jointly and different combinations can lead to the same outcome. PLS-SEM follows a net-effect logic and assumes symmetric relationships, which may be limited in capturing equifinal pathways and conjunctural causation—for example, high co-creation intention may emerge from distinct configurations such as “high empowerment with strong institutional interaction” versus “high empowerment with strong resident interaction,” and the absence of a single condition does not necessarily imply the absence of the outcome [82].

To address this limitation and strengthen the explanatory payoff of the empirical analysis, the study further applied fuzzy-set qualitative comparative analysis (fsQCA) to explore how different configurations of interaction conditions and AI-enabled stimuli jointly produce high (and low) Co-creation Intention (CI). By integrating fsQCA, we adopt a configurational perspective capable of identifying multiple sufficient pathways, asymmetry, and conditional complementarities that may be overlooked in SEM’s symmetric estimation [83]. This dual-method strategy provides a more holistic and ecologically valid explanation of how host–guest interaction types, empowerment, and AI-enabled platform conditions collectively shape tourists’ co-creation intention in urban tourism settings.

### 5.1 Variable selection and calibration

In the fsQCA analysis, this study selected Tourist–Government Interaction (TGI), Tourist–Resident Interaction (TRI), Customer Empowerment (CE), and the two dimensions of AI technology stimuli (AITS)—Passion (P) and Usability (U)—as antecedent conditions, with Co-creation Intention (CI) as the outcome. This variable set aligns with the proposed “interaction type → empowerment → co-creation intention” logic and enables a configurational assessment of how multiple conditions jointly lead to high (or low) co-creation intention.

Following established fsQCA guidelines [84], we applied the direct method of calibration to transform raw Likert-scale scores into fuzzy-set membership scores ranging from 0 to 1. Specifically, three qualitative anchors were set for each variable: the 95th percentile as the threshold for full membership, the 50th percentile as the crossover point, and the 5th percentile as the threshold for full non-membership, providing a stricter set definition and reducing ambiguity around the middle range (Table 18).These thresholds were then implemented using the Calibrate function in the fsQCA software to generate calibrated set memberships for subsequent necessary-condition and sufficiency (configurational) analyses.

**Table 18:** Calibration anchors.

	TGI	TRI	CE	P	U	AITS	CI
Full membership	6.25	6.25	6.25	6.25	6.25	6	6.25
Crossover point	4.75	4.75	4.75	4.75	5	4.875	5
Full non-membership	2.5	3.075	3.25	3.25	3.25	3.625	3.25

The calibration anchors for each variable were set at the 95th, 50th, and 5th percentiles.

### 5.2 Necessity analysis

Before conducting the configurational analysis, a necessity analysis was performed to determine whether any single condition was necessary for achieving high or low Co-creation Intention (CI). A condition is considered necessary if its consistency score exceeds 0.90 and it demonstrates a significant level of coverage [85].

The necessity analysis was applied to all relevant antecedent conditions. As shown in Table 19, none of the conditions met the 0.90 consistency threshold, indicating that no individual antecedent condition is necessary for either high or low Co-creation Intention (CI). Although some conditions, such as TGI, CE, and P, show relatively high consistency scores for High CI, their coverage values and consistency for Low CI were insufficient to establish necessity. Consequently, the results suggest that the outcome of Co-creation Intention (CI) is not solely dependent on any single condition but rather the combination of multiple factors, as further explored in the subsequent configurational analysis.

**Table 19:** Necessity Analysis.

	High CI		Low CI	
	Consistency	Coverage	Consistency	Coverage
TGI	0.727340	0.640500	0.672710	0.629660
Low TGI	0.579450	0.624860	0.615920	0.705970
TRI	0.687180	0.642300	0.628980	0.624870
Low TRI	0.598660	0.602870	0.639950	0.684980
CE	0.689570	0.700600	0.551590	0.595660
Low CE	0.602020	0.558130	0.722750	0.712210
P	0.647170	0.616380	0.649040	0.657030
Low P	0.639900	0.631720	0.621050	0.651680
U	0.603630	0.599100	0.631980	0.666690
Low U	0.664170	0.629340	0.619970	0.624410
AITS	0.606930	0.603420	0.638400	0.674640
Low AITS	0.672750	0.636420	0.624720	0.628160

### 5.3 Configurational analysis

Configurational analysis is used to reveal how multiple antecedent conditions jointly generate an outcome through equifinality (i.e., multiple pathways can lead to the same outcome) and causal asymmetry (i.e., the configurations leading to high versus low outcomes are not simply mirror opposites) [86]. Following standard fsQCA procedures, this study standardized the truth table using a consistency threshold of 0.80, a minimum case frequency of 3, and a PRI threshold of 0.70. As shown in Table 20, the configurational‌‌ solution for High Co-creation Intention (High CI) demonstrates an acceptable model fit (solution consistency = 0.83; solution coverage = 0.51), whereas the solution for Low Co-creation Intention (Low CI) shows higher consistency (0.86) with moderate coverage (0.40). These results indicate that high and low co-creation intention are produced by different causal recipes, rather than a simple mirror-image relationship.

**Table 20:** Configurational analysis‌‌.

	High CI				Low CI		
	1	2	3	4	5	6	7
TGI	⊗	⊗	×	×	⊗		⊗
TRI		×	×	⊗	⊗	⊗	
CE	×	×	×	×	⊗	⊗	⊗
P	⊗		×	×		×	×
U	⊗	⊗		⊗	×	×	×
AITS	⊗	⊗	×	⊗	×	×	×
Raw Coverage	0.29	0.28	0.37	0.25	0.31	0.33	0.33
Unique Coverage	0.02	0.01	0.14	0.02	0.02	0.04	0.05
Consistency	0.86	0.88	0.87	0.90	0.88	0.88	0.89
Solution Coverage	0.51				0.40		
Solution Consistency	0.83				0.86		

“×” indicates the presence of a high level of a core condition. “⊗” indicates the presence of a low level (negation) of a core condition.

**Pathways to High Co-creation Intention (High CI)**

**Pathway 1** indicates that tourists can exhibit high CI (consistency = 0.86, unique coverage = 0.02) when Customer Empowerment (CE) is present as a core condition, even when Tourist–Government Interaction (TGI), Passion (P), Usability (U), and AI technology stimuli (AITS) are at low levels (core negations). This pathway suggests that empowerment is a decisive psychological trigger: once tourists feel empowered, co-creation intention can emerge even in relatively “low-tech” or weakly stimulated contexts, implying that empowering experiences can compensate for limited institutional interaction and perceived AI support.

**Pathway 2** shows that High CI is achieved (consistency = 0.88, unique coverage = 0.01) when TRI and CE are both core present, despite low TGI, low U, and low AITS. This reflects a typical community-embedded mechanism: strong tourist–resident engagement, coupled with a heightened sense of empowerment, can generate co-creation intention even without strong AI-enabled platform stimuli. In other words, relational embeddedness and empowerment jointly substitute for technology under certain conditions.

**Pathway 3** reveals the most distinctive High-CI recipe (consistency = 0.87, unique coverage = 0.14) where TGI, TRI, CE, P, and AITS are all core present. This configuration represents an “AI-amplified synergy” pathway: co-creation intention is strongest when tourists are empowered and simultaneously embedded in both institutional (government) and social (resident) interactions, while AI-enabled stimuli and motivational engagement (passion) reinforce the translation of interaction into co-creation intention. Notably, this pathway provides the largest unique explanatory contribution among High-CI solutions, highlighting the value of a configurational lens: technology matters most when it aligns with multi-actor interaction and empowerment, rather than operating as a stand-alone driver.

**Pathway 4** indicates that High CI can also occur (consistency = 0.90, unique coverage = 0.02) when TGI, CE, and P are core present, even under low TRI, low U, and low AITS. This suggests a more “institution-centered” route: empowered tourists may develop co-creation intention primarily through formal or governance-related interaction channels, while resident interaction and perceived AI usability/stimuli are not necessary and may even be absent in the core recipe. Substantively, this pathway implies that institutional engagement combined with a motivated psychological state can still produce high co-creation intention, even without strong community embeddedness or strong perceived AI support.

**Pathways to Low Co-creation Intention (Low CI)**

**Pathway 5** shows Low CI (consistency = 0.88, unique coverage = 0.02) when low TGI, low TRI, and low CE co-occur, even though U and AITS are high. This indicates that a technology-rich setting does not compensate for weak interaction and disempowerment: when tourists lack meaningful interaction opportunities and do not feel empowered, co-creation intention remains low even if platforms appear usable and AI-stimulating.

**Pathway 6** (consistency = 0.88, unique coverage = 0.04) and **Pathway 7** (consistency = 0.89, unique coverage = 0.05) further reinforce this insight: Low CI emerges when low CE combines with low interaction (particularly low TRI in Pathway 6 and low TGI in Pathway 7), despite the presence of high P, high U, and high AITS. These pathways suggest that even when AI-enabled platforms stimulate engagement motivation and provide functional usability, co-creation intention does not materialize without empowerment, indicating a clear boundary of “technology-first” approaches.

### 5.4 Robustness check

When the consistency threshold was increased from 0.80 to 0.85 while keeping all other settings unchanged, the solutions remained the same, indicating that the results are robust.

## 6 Discussion

### 6.1 Findings from the SEM analysis

**First,** the SEM results show that neither Tourist–Government Interaction (TGI) nor Tourist–Resident Interaction (TRI) directly predicts Co-creation Intention (CI) (H1a and H1b not supported). This finding is theoretically meaningful because it challenges a simplified assumption that more host–guest interaction will automatically generate stronger value co-creation intentions. In the context of smart urban tourism, not all interaction is inherently transformative. Many forms of contact remain informational, procedural, or situational—for example, route inquiries, service consultation, routine feedback, or brief exchanges with local actors. Although such interactions may increase exposure to destination resources, they do not necessarily alter tourists’ perceived role in the value creation process. In other words, interaction alone does not guarantee a shift from passive service consumption to active contribution. This finding is consistent with prior arguments that interaction lacking emotional depth, participatory significance, or perceived redistribution of influence is unlikely to trigger deeper involvement [87]. At the same time, the present study extends this line of thinking by showing that the critical issue is not whether interaction occurs, but whether that interaction enables tourists to perceive themselves as consequential actors within the destination service system. Thus, the non-significant direct effects should not be interpreted as evidence that host–guest interaction is unimportant; rather, they suggest that its influence is conditional and mechanism-dependent. For tourism managers and policymakers, this implies that simply increasing contact points between tourists and local actors may be insufficient unless those interactions are designed in ways that make participation meaningful, visible, and influential.

**Second,** Customer Empowerment (CE) emerges as the pivotal psychological mechanism through which interaction becomes behaviorally consequential. Both interaction types significantly enhance CE (TGI → CE: β = 0.340, p < 0.001; TRI → CE: β = 0.269, p < 0.001), and CE in turn strongly predicts CI (CE → CI: β = 0.363, p < 0.001). Bootstrapping further confirms significant indirect effects via CE for both TGI and TRI, while the direct paths from interaction to CI remain non-significant, indicating an indirect-only (full mediation) pattern. This result is important because it clarifies how host–guest interaction translates into co-creation intention: not by direct exposure to contact itself, but by strengthening tourists’ perceived voice, influence, and participatory legitimacy. In this sense, empowerment should not be viewed as an auxiliary attitudinal variable, but as the core psychological mechanism that converts interaction into intention. This interpretation is consistent with Shin et al. (2020) [58], yet the present study goes further by locating empowerment more explicitly within a host–guest interaction framework and by showing that both institutional and affective interaction forms require this psychological conversion before co-creation intention can emerge. The contribution of the model therefore lies not in demonstrating large direct effects, but in identifying why interaction frequently fails to generate co-creation unless tourists feel able to matter in the process. Although the explained variance of CI is moderate (R^2^ = 0.258), this level of explanatory power is theoretically informative rather than problematic, as it reflects the fact that co-creation intention in urban tourism is not a purely additive outcome but a conditional and psychologically mediated response [88]. More importantly, the findings indicate that destinations seeking to stimulate co-creation should not focus solely on increasing interaction opportunities; they should also create participatory arrangements through which tourists can express preferences, influence service processes, and perceive their involvement as consequential. This has direct implications for smart tourism governance, suggesting that empowerment-oriented participation design may be more effective than contact-oriented engagement strategies alone.

**Third,** AI technology stimuli (AITS) exhibits a distinctly context-dependent moderating role, thereby clarifying when technology adds incremental value to host–guest interaction. In the tourist–government context, AITS significantly strengthens the empowerment effect of institutional interaction (AITS × TGI → CE: β = 0.213, p < 0.001) and also strengthens the relationship between TGI and CI (AITS × TGI → CI: β = 0.171, p < 0.001), supporting H4a and H5a. This pattern suggests that AI-enabled features—such as faster responsiveness, transparent process feedback, personalized service support, and efficient handling of requests—can enhance the perceived usefulness and procedural significance of institutional interaction. Under such conditions, tourists are more likely to view government-linked interaction channels not merely as service tools, but as effective interfaces through which their input can be acknowledged and acted upon. This interpretation is in line with prior studies suggesting that AI can enhance perceived reliability and trust in smart governance settings [66] and strengthen confidence and perceived agency in co-creation processes [89]. However, the present study refines this view by showing that the contribution of AI is not universal; rather, it is strongest when interaction is embedded in institutional and procedural settings that rely on information processing, coordination, and responsiveness. For tourism authorities and platform designers, this implies that investment in AI is most likely to generate value when it is embedded in governance-oriented participation systems—for example, in smart feedback channels, public service interfaces, and participatory destination management tools.

By contrast, AITS does not significantly moderate the TRI → CE or TRI → CI relationships (H4b and H5b not supported), suggesting that the role of AI is more limited in affective and culturally embedded interaction contexts. Tourist–resident interaction appears to rely more heavily on authenticity, emotional resonance, social warmth, and cultural identification, where co-creation is grounded in interpersonal trust and social embeddedness rather than procedural efficiency [90, 91]. In such contexts, AI may support peripheral functions, but it does not necessarily strengthen the core relational qualities that make resident interaction meaningful. This finding is theoretically important because it highlights an asymmetry in the value of intelligent technologies across interaction types. It suggests that AI should not be treated as a universally beneficial enhancer of all host–guest interaction, but as a differentiated enabler whose effect depends on the relational structure of the interaction itself [17]. This interpretation is also consistent with broader evidence that psychologically rich and emotionally evocative experiences often generate distinctive affective and restorative responses, which are not easily reducible to informational efficiency or technological facilitation alone [92]. In other words, AI functions more effectively as an amplifier of institutional coordination than as a substitute for human-to-human relational depth. This distinction advances current debates in smart tourism by showing that technological sophistication does not automatically translate into stronger co-creation capacity across all social settings. Instead, technology appears to add value where participation is structured, procedural, and system-mediated, but it is less able to reproduce the emotional nuance, mutual recognition, and lived authenticity embedded in resident-based interaction. Practically, this suggests that destination managers should avoid assuming that digital intelligence can replace the relational foundations of community-based tourism. Rather, AI and human interaction should be treated as complementary but not interchangeable mechanisms in the design of smart urban tourism experiences.

### 6.2 Fuzzy-set qualitative comparative analysis (fsQCA)

The fsQCA findings complement the SEM results by showing that co-creation intention in smart urban tourism is not generated through a single linear causal path, but through multiple contextually contingent combinations of conditions. This is theoretically important because it moves the analysis beyond net-effect logic and demonstrates that the same outcome may be achieved through different interactional and technological arrangements. Consistent with the principles of equifinality and causal asymmetry, high and low co-creation intention are not mirror images of one another, nor are they driven by a single dominant predictor. Instead, they emerge from different conjunctural “recipes,” each reflecting a distinct pattern of interaction, empowerment, and AI-related support. In this sense, the fsQCA results deepen the study’s explanatory logic by showing that value co-creation intention is not simply the additive result of stronger interaction or higher technology exposure, but the outcome of a specific fit among psychological, relational, and technological conditions.

Across all solutions, Customer Empowerment (CE) emerges as the most structurally consequential condition. It appears as a core present condition in all high-CI pathways, whereas its absence systematically anchors the low-CI configurations. This finding reinforces the SEM-based mediation result and further strengthens the study’s main theoretical argument: empowerment is not merely a statistically significant mediator, but a configurationally central condition that determines whether interaction and technology can be converted into co-creation intention. Put differently, empowerment functions as a form of participation threshold. When tourists perceive themselves as capable of expressing preferences, influencing processes, and participating meaningfully, different interaction and technology conditions can still lead to co-creation intention through alternative routes. When such empowerment is absent, however, even otherwise favorable contextual conditions become insufficient. This extends prior interaction-based research by suggesting that the decisive issue is not only whether resources are available, but whether tourists feel entitled and able to mobilize them within the destination service system.

For high co-creation intention, the configurational results identify four distinct pathways, each revealing a different way in which interaction and AI technology stimuli can operate. Pathway 1 indicates that high CI can emerge even in a relatively “low-tech/low-stimulus” context when CE is core present despite low levels of Tourist–Government Interaction (TGI), Passion (P), Usability (U), and AI technology stimuli (AITS). The theoretical implication of this pathway is that empowerment may compensate for weak institutional support and limited technological facilitation. In other words, an internalized sense of agency can itself become a sufficient activation condition for co-creation intention even when external enabling factors are relatively weak [93]. Pathway 2 reveals a community-embedded route in which CE and Tourist–Resident Interaction (TRI) jointly compensate for low TGI, low U, and low AITS. This suggests that relational embeddedness and resident-based interaction can sustain co-creation even under weak technological and institutional conditions, provided that tourists feel meaningfully empowered. This pathway is especially important because it refines the role of AI in the model: rather than being a universal enabler, AI appears non-essential in interaction settings where social trust, local connection, and participatory confidence already provide a strong basis for co-creation.

By contrast, Pathway 3 represents the clearest AI-amplified synergy configuration, in which TGI, TRI, CE, P, and AITS are all core present and together produce high CI with the largest unique explanatory contribution. This pathway is theoretically distinctive because it shows that technology is most consequential not as an isolated driver, but as an amplifier operating within an already favorable interactional and motivational structure. In this configuration, AI does not substitute for interaction; rather, it intensifies the extent to which multi-actor engagement is translated into co-creation intention. This interpretation is consistent with recent evidence suggesting that the value of AI in tourism depends less on simple technological adoption than on deeper user engagement, which enhances smart experiences, emotional involvement, and downstream behavioral responses [94]. It also aligns with the study’s central argument that AI stimuli become behaviorally effective only when they fit the relational structure of the interaction context. Pathway 4 adds further nuance by identifying an institution-centered route in which high CI is achieved when TGI, CE, and P are core present, even under low TRI, low U, and low AITS. This suggests that co-creation intention can also be activated through governance-related participation channels when tourists are both motivated and empowered, even if community embeddedness and strong AI usability are not central ingredients. Taken together, the four high-CI pathways show that empowerment is the common foundation, while the dominant route to co-creation depends on whether participation is anchored more strongly in resident interaction, institutional interaction, or AI-supported synergy.

For low co-creation intention, the fsQCA results provide a particularly important boundary insight for smart tourism governance. Pathway 5 shows that even when U and AITS are high, low CI still emerges when low TGI, low TRI, and low CE occur together. This demonstrates that a technology-rich environment cannot compensate for weak interaction opportunities and low empowerment. Pathways 6 and 7 reinforce this conclusion by showing that low CI persists even in the presence of high P, high U, and high AITS when low CE combines with low interaction—particularly low TRI in Pathway 6 and low TGI in Pathway 7. These findings reveal a critical asymmetry: while AI stimuli and usability can facilitate engagement under empowerment-consistent configurations, they do not override disempowerment. This is one of the most important theoretical implications of the fsQCA results. It suggests that technology-first approaches in smart tourism may be fundamentally limited if they are not accompanied by meaningful interaction structures and participatory conditions. In other words, digital sophistication alone does not guarantee co-creation. Without empowerment, tourists may remain users of intelligent services, but not active contributors to destination value formation.

Overall, the fsQCA results sharpen and extend the study’s central claim. Whereas the SEM results demonstrate the average net effects of interaction, empowerment, and AI stimuli, the fsQCA results reveal how these conditions must be configured in order for co-creation intention to emerge. Together, they show that co-creation intention in smart urban tourism is not a product of any single factor operating independently, but a contextually fitted outcome generated through different combinations of empowerment, relational embedding, institutional support, and AI-related facilitation. This not only strengthens the study’s theoretical contribution, but also carries practical implications for destination stakeholders and policymakers: strategies aimed at increasing co-creation should not rely solely on technological upgrading, but should instead build interaction systems in which tourists are empowered to participate meaningfully through pathways that fit the specific relational context of the destination

### 6.3 Integration of PLS-SEM and fsQCA results

Integrating PLS-SEM and fsQCA provides a more comprehensive explanation of how host–guest interaction types shape tourists’ co-creation intention in smart urban tourism. Rather than offering redundant evidence, the two methods illuminate different dimensions of the same phenomenon. PLS-SEM identifies the symmetric net-effect structure of the model and clarifies the primary psychological mechanism underlying co-creation intention. Specifically, it shows that Customer Empowerment (CE) is the most proximate driver of Co-creation Intention (CI), that both Tourist–Government Interaction (TGI) and Tourist–Resident Interaction (TRI) significantly enhance CE, and that neither interaction type exerts a statistically significant direct effect on CI. This pattern supports an indirect-only (full mediation) mechanism, indicating that host–guest interaction influences co-creation primarily by cultivating a sense of agency, influence, and participatory legitimacy. In addition, the moderation tests reveal clear contextual heterogeneity: AI technology stimuli (AITS) significantly strengthens the effects of TGI on both CE and CI, but does not significantly moderate TRI-related relationships. In line with prior evidence suggesting that the benefits of digital systems are often participation-contingent rather than universally distributed [95], the PLS-SEM results indicate that AI functions less as a general enhancer of all interaction and more as a conditional amplifier in institutional participation contexts.

By contrast, fsQCA contributes a configurational perspective that reveals how co-creation intention is generated through different combinations of conditions rather than through a single dominant path. This is theoretically important because it demonstrates that the logic of co-creation in smart urban tourism is not purely linear or additive. Instead, high and low CI emerge from multiple conjunctural “recipes,” consistent with the principles of equifinality and causal asymmetry. The configurational findings deepen the PLS-SEM results in three ways. First, they reinforce the centrality of empowerment by showing that CE appears as a core present condition across all high-CI pathways, whereas low CE anchors the low-CI solutions. This suggests that empowerment is not only a mediator in the statistical sense, but also a structural gateway that determines whether interaction and technology can be translated into co-creation intention. Second, fsQCA clarifies why some net effects in PLS-SEM appear modest or conditional: interaction and technology do not always matter independently, but often become consequential only when aligned with other enabling conditions. The “AI-amplified synergy” pathway, in which TGI, TRI, CE, Passion (P), and AITS are all core present, illustrates that AI becomes most behaviorally meaningful when embedded in a broader interactional and motivational structure, rather than operating as a stand-alone driver. Third, fsQCA establishes a clear theoretical and practical boundary for technology-first approaches. Several low-CI configurations show that even high AITS and high Usability (U) cannot compensate for weak interaction and low empowerment, thereby demonstrating that platform sophistication alone is insufficient to activate co-creation when participatory conditions are absent.

Taken together, the two methods converge on a shared conclusion while also contributing different forms of explanatory value. PLS-SEM explains the average causal chain by identifying the key mediating and moderating relationships, whereas fsQCA reveals the multiple, asymmetric pathways through which those relationships are activated, combined, or neutralized in specific contexts. More importantly, their integration leads to a stronger theoretical insight: co-creation intention in smart urban tourism is not triggered by interaction per se, nor by technology per se, but by interaction that is psychologically empowering and technologically context-fitted. In this sense, empowerment constitutes the core conversion mechanism, while AI serves as a differentiated enabler whose value depends on whether it is aligned with the relational structure of the interaction context. This integrated understanding extends prior research by showing that value co-creation should be understood not only as a mediated process, but also as a configurationally assembled outcome shaped by the interplay of interaction, empowerment, and technology.

From a practical perspective, the integrated results also imply that destination stakeholders and policymakers should avoid relying on one-dimensional strategies. Simply increasing host–guest contact or investing in more advanced digital systems is unlikely to generate co-creation on its own. Instead, smart tourism policies and destination management practices should focus on building participation systems in which tourists are empowered to contribute meaningfully and in which AI tools are embedded in ways that fit the institutional or relational nature of the interaction setting. Such an approach is more likely to produce sustainable and context-sensitive forms of value co-creation in urban tourism.

## 7 Conclusion

### 7.1 Theoretical implications

This study contributes to the smart tourism and value co-creation literature in three ways. First, it clarifies the psychological mechanism linking host–guest interaction to co-creation intention by identifying customer empowerment as a key mediating factor. The results show that both tourist–government interaction and tourist–resident interaction enhance co-creation intention mainly by strengthening tourists’ perceived autonomy, participation, and influence, rather than through direct effects alone. This finding extends prior research by showing that interaction contributes to co-creation only when it is experienced as empowering.

Second, this study refines the understanding of artificial intelligence in smart tourism by showing that its role is context-dependent rather than universally positive. The results indicate that artificial intelligence technology stimuli strengthen the effects of tourist–government interaction on both customer empowerment and co-creation intention, whereas their moderating effects are not supported in tourist–resident interaction. This suggests that artificial intelligence is more effective in institutional and platform-mediated settings than in socially embedded and culturally oriented interaction contexts.

Third, by combining structural equation modeling and fuzzy-set qualitative comparative analysis, this study provides both symmetric and configurational evidence on the formation of co-creation intention. While the structural model identifies the net effects of interaction, empowerment, and technology conditions, the configurational results show that high co-creation intention can emerge through multiple pathways. Together, these findings offer a more nuanced understanding of how interaction, empowerment, and artificial intelligence jointly shape co-creation in smart urban tourism.

### 7.2 Practical implications

The findings offer several practical implications for local governments, platform operators, and destination managers. First, smart tourism governance should focus not only on increasing interaction opportunities but also on strengthening tourists’ sense of empowerment. Participation mechanisms, feedback channels, and visible response processes can help tourists feel that their involvement is meaningful and influential.

Second, destination managers should pay greater attention to the social and cultural value of tourist–resident interaction. Resident-led activities, community-based experiences, and participatory cultural programs can create stronger emotional connection and encourage tourists to move from passive consumption to active contribution.

Third, artificial intelligence should be deployed in a context-sensitive way. In tourist–government interaction, artificial intelligence can improve responsiveness, transparency, and service efficiency, thereby amplifying empowerment and co-creation intention. In tourist–resident interaction, however, technology should mainly serve as supportive infrastructure, such as reducing communication barriers and improving coordination, without replacing the authenticity and emotional depth of human exchange.

Overall, this study shows that value co-creation in smart urban tourism is shaped not simply by more interaction or more technology, but by whether interaction and technology together enhance tourists’ sense of participation and influence.

### 7.3 Limitations and further research

This study has several limitations. First, the quantitative data were collected through a non-probability online survey, which may introduce selection bias and limit the generalizability of the findings. In addition, the measures relied on single-source self-reported data, which may be affected by social desirability and common method bias. Future studies could use probability-based or quota sampling, conduct multi-site comparisons, and combine survey data with behavioral or platform-use records.

Second, the study examined tourists’ perceptions at one point in time and therefore could not capture changes in empowerment or co-creation intention across different travel stages. Future research could adopt longitudinal or experience-sampling designs to examine how interaction experiences shape customer empowerment and co-creation over time.

Third, the role of artificial intelligence technology stimuli was examined under the current development level of smart tourism platforms. As artificial intelligence becomes more deeply embedded in tourism services, its boundary effects may change. Future studies could compare different destinations and platform systems to further examine how technological development reshapes the relationships among interaction, empowerment, and co-creation.
